# MoMaf1 Mediates Vegetative Growth, Conidiogenesis, and Pathogenicity in the Rice Blast Fungus *Magnaporthe oryzae*

**DOI:** 10.3390/jof9010106

**Published:** 2023-01-12

**Authors:** Bin Qian, Lingyuan Guo, Chi Song, Hong Ji

**Affiliations:** College of Biology and Food Engineering, Changshu Institute of Technology, Changshu 215500, China

**Keywords:** *Magnaporthe oryzae*, RNA polymerase (Pol) III, transfer RNA (tRNA), pathogenicity, cell wall integrity

## Abstract

In eukaryotes, Maf1 is an essential and specific negative regulator of RNA polymerase (Pol) III. Pol III, which synthesizes 5S RNA and transfer RNAs (tRNAs), is suppressed by Maf1 under the conditions of nutrient starvation or environmental stress. Here, we identified *M. oryzae* MoMaf1, a homolog of ScMaf1 in budding yeast. A heterogeneous complementation assay revealed that MoMaf1 restored growth defects in the Δ*Scmaf1* mutant under SDS stress. Destruction of *MoMAF1* elevated 5S rRNA content and increased sensitivity to cell wall agents. Moreover, the Δ*Momaf1* mutant exhibited reduced vegetative growth, conidiogenesis, and pathogenicity. Interestingly, we found that MoMaf1 underwent nuclear-cytoplasmic shuffling, through which MoMaf1 accumulated in nuclei under nutrient deficiency or upon the interaction of *M. oryzae* with rice. Therefore, this study can help to elucidate the pathogenic molecular mechanism of *M. oryzae*.

## 1. Introduction

Protein synthesis requires coding and noncoding RNAs, and the synthesis process is regulated by multiple growth conditions. The transcription of protein synthesis involves regulation of all three RNA polymerases: ribosomal RNAs are mostly transcribed by polymerase I (Pol I), ribosomal protein-coding mRNAs are transcribed by Pol II, and 5S rRNA and tRNAs are transcribed by Pol III [1,2].

In eukaryotes, dividing cells always contain more tRNAs and 5S rRNAs than resting cells, but the presence of too many tRNAs often has adverse effects in cells [3]. For example, excess tRNAs in mammalian cells can increase the risk of carcinogenesis [3,4]. Pol III repressor, Maf1, is required for the attenuation of the Pol III system in unfavorable growth conditions: nutrient starvation, oxidative stress, and cell wall stress [5].

In yeast, Maf1 is a phosphoprotein, and under favorable conditions, Maf1 is phosphorylated and mostly cytoplasmic, allowing Pol III transcription [6]. Phosphorylation by TOR-regulated kinase Sch9 and protein kinase A (PKA), and nuclear export by Msn5 [7,8,9], are important for Maf1 maintaining its cytoplasmic localization. Upon nutrient deprivation, Maf1 is dephosphorylated and moves into the nucleus, where it binds Pol III and represses tRNA transcription [7]. To activate tRNA transcription again, casein kinase II phosphorylates Maf1 to promote the dissociation of Maf1 from Pol III [10].

The rice blast fungus *Magnaporthe oryzae* causes rice blast, a devastating global disease, and is also a widely adopted model organism for studying plant–pathogen interactions [11]. *M. oryzae* is a hemi-biotrophic fungus undergoing an initial biotrophic stage prior to switching to a necrotrophic stage that promotes plant cell death [12]. During the infection process, how the fungus adapts to the nutrient starvation stress in rice cells is always a key question. Studies have revealed that the modulation of TOR signaling pathway was important for *M. oryzae* to adapt to nutrient starvation [13]. Under nutrient limitations, TOR signaling is inhibited and leads to the separation of Tap42 and MoPpe1 [13,14]. The released MoPpe1 promotes the utilization of non-preferred nutrients (such as nitrate) and activates the CWI pathway to regulate the virulence of *M. oryzae* [13,14]. In this study, we established a link between infection and protein synthesis by monitoring the localization of MoMaf1, which is essential for the pathogenicity of *M. oryzae.*

## 2. Materials and Methods

### 2.1. Fungal Strains and Culture Conditions

All tested strains were cultured on CM at 28 °C, and the wild-type Guy11 strain was used for transformation in this study. Vegetative growth of Δ*Momaf1* mutant, complemented strain*,* and Guy11 was measured on complete medium (CM) (50 mL 20× nitrate salts, 1 mL trace elements, 10 g glucose, 2 g peptone, 1 g yeast extract, 1 g casamino acids, 1 mL vitamin solution, 15 g agar in 1 L distilled water), minimal medium (MM) (6 g NaNO_3_, 0.52 g KCl, 0.152 g MgSO_4_·7 H_2_O, 1.52 g KH_2_PO_4,_ 0.01 g VB1, 1 mL trace elements, 10 g glucose in 1 L distilled water), oatmeal agar medium (OM) (30 g oatmeal and 10 g agar in 1 L distilled water), and straw decoction and corn agar medium (SDC) (100 g straw, 40 g corn powder, 15 g agar in 1 L distilled water) for 7 days as described previously [15]. 

For conidiation, mycelial blocks were inoculated on (SDC) at 28 °C for 7 days in the dark, followed by 3 days of illumination under fluorescent light [16]. Mycelia were harvested from liquid CM and then used for DNA and protein extraction.

### 2.2. Quantification of Gene Expression during Different Phases

Total RNA samples were extracted from mycelia, conidia, and infected rice leaves, respectively. Reverse transcriptase HiScript III RT SuperMix for qPCR (Vazyme Biotech Co., Ltd., Nanjing, China) was used to prepare cDNA. qRT-PCR was run on an Applied Biosystems (Foster City, CA, USA) 7500 Real-time PCR System with SYBR Premix Ex Taq (Vazyme Biotech Co., Ltd., Nanjing, China). The 2^−ΔΔCT^ method [17,18] was used to calculate the relative quantification of each transcript with the *M. oryzae* actin gene as the internal control. The experiment was repeated three times with three biological replicates.

### 2.3. Nucleic Acid Manipulation and qRT-PCR

Total RNA was extracted from each sample using the RNeasy mini kit from Qiagen (Shanghai, China). RNA were manipulated using standard procedures.

For qRT-PCR, 5 μg of total RNA was reverse transcribed into cDNA. qRT-PCR was performed following previously established procedures [19] and the relative abundance of target transcripts was normalized to that of the actin gene (MGG_03982). Three independent replicates were performed for each experiment; the qRT-PCR primers used were listed in Table S1. Data analysis was performed using the delta delta-CT (2^−ΔΔCT^) method [20,21].

### 2.4. The Complementation of S. cerevisiae Δmaf1 Mutant

The full-length cDNA of *MoMAF1* was amplified with primers (Supporting Information Table S1), digested with XbaI and SacI, and then cloned into the yeast expression vector pYES2 (Invitrogen). After verification by sequencing and selection on SD medium lacking uracil, the *MoMAF1*-pYES2 vector was transformed into the yeast Δ*Momaf1* mutant (BY4741, DYRE122C). Yeast strains were cultured on YPD medium and diluted to an OD600 of 0.1, after which 5 µL of 10-fold serial dilutions grew on SD-Met-Leu-His-Ura (galactose) and SD-Met-Leu-His-Ura supplemented with 0.1% SDS plates at 30 °C for 4 days before being photographed.

### 2.5. MoMAF1 Gene Deletion and Complementation

The Δ*Momaf1* mutants were generated by the one-step gene replacement strategy. Two 1.0 kb fragments flanking the targeted gene were PCR amplified with primer pairs (Supplementary Table S1). The two 1.0 kb fragments were ligated to the two ends of the hygromycin resistance cassette (*HPH* 1.4 kb) by overlap PCR to form a 3.4 kb fragment. Then, the 3.4 kb fragment was transformed into Guy11 protoplasts by transformation [22]. The putative mutants were screened by PCR after 7–10 days of incubation at 28 °C and further confirmed by Southern blot analysis.

To generate a complementary pYF11-*MoMAF1*-GFP fusion construct, the gene sequence containing the *MoMAF1* gene and 1.5 kb native promoter was amplified by PCR. Yeast strain XK125 was cotransformed with this sequence and the *Xho*I-digested pYF11 plasmid (containing a bleomycin resistance gene and GFP sequence) by the yeast gap repair approach. Then, obtained yeast plasmid was expressed in *E*. *coli*. To generate the complementary strain, the pYF11-*MoMAF1* construct containing the bleomycin resistance gene for the *M. oryzae* transformants screen was introduced into the Δ*Momaf1* mutant.

### 2.6. Conidial Germination and Appressorium Formation

Conidial germination and appressorium formation were measured on a hydrophobic surface. Conidial suspensions of 25 μL (5 × 10^4^ spores/mL) were dropped onto a hydrophobic surface and placed in a humidified box at 28 °C. The appressorium formation rate was counted at 24 h postinoculation (hpi) under a microscope, and more than 200 appressoria were counted for each strain.

### 2.7. Host Penetration and Pathogenicity Assay

For the spraying assay, two-week-old rice seedlings (*O. sativa* cv. CO39) were sprayed with 5 mL of the conidial suspension and kept in a growth chamber at 28 °C with high humidity (>80%) in the dark for the first 24 h, followed by incubation under a 12 h light:12 h dark cycle for 7 days. For the barley infection assay, 7-day-old barley leaves were inoculated with three droplets (25 μL) of the conidial suspension, and photographs were taken on day 5 after infection. Each experiment was repeated at least three times. To assess rice sheath penetration and invasive hyphal expansion, the conidial suspension (1 × 10^5^ spores/mL) was inoculated into the sheaths. After incubation at 28 °C for 30 h, the sheath cuticle cells were observed under a Zeiss Axio Observer A1 inverted microscope.

### 2.8. Western Blot Analysis of Protein Phosphorylation

The Δ*Momaf1* mutant and wild-type strains were cultured in liquid CM for 2 days and then harvested, and 1 mL of protein lysis buffer and 10 µL of protease inhibitor cocktail (Sangon, Shanghai, China) were added. After vortexing and homogenization, the lysate was centrifuged at 12,000 rpm for 10 min at 4 °C. Then, 200 μL of the supernatant was mixed with 50 μL loading buffer and boiled for 5 min. Obtained proteins were separated on SDS–PAGE gels and transferred onto a polyvinylidene fluoride membrane using a Bio-Rad blotting apparatus. The intensity of the phosphorylated Mps1 signal was detected by the addition of an anti-phospho-p44/42 MAP kinase antibody (Cell Signaling Technology, Boston, MA, USA), with an anti-MAPK1 antibody (N-terminal anti-Mpk1) used as a control.

### 2.9. Chitin (N-acetylglucosamine, GlcNAc) Content Assay

The chitin (*N*-acetylglucosamine, GlcNAc) content was analyzed as follows. First, mycelial samples were freeze-dried, and then 5 mg of the dried mycelia was resuspended in 1 mL of 6% KOH and heated at 80 °C for 90 min. The samples were centrifuged (16,000× *g*, 10 min), and the pellets were washed with PBS over three cycles of centrifugation and resuspension (16,000× *g*, 10 min) before the final suspension in 0.5 mL of McIlvaine’s buffer (pH 6). An aliquot of 100 mL (13 units) of *Streptomyces plicatus* chitinase (Sigma, St. Louis, MO, USA) was added, and the mixture was incubated for 16 h at 37 °C with gentle mixing; 100 mL samples were then combined with 100 mL of 0.27 M sodium borate (pH 9) and heated for 10 min at 100 °C with the final addition of 1 mL of freshly diluted (1:10) Ehrlich’s reagent (10 g of *p*-dimethylaminobenzaldehyde in 1.25 mL of HCl and 8.75 mL of glacial acetic acid). After incubation at 37 °C for 20 min, 1 mL of the sample was transferred to a 2.5 mL plastic cuvette (Greiner, Frickenhausen, Germany), and the absorbance at 585 nm was recorded. Standard curves were prepared with GlcNAc (Sigma). The experiment was repeated three times.

### 2.10. The Observation of Subcellular Localization

To observe the subcellular localization of MoMaf1, we fused MoMaf1 with a GFP tag and a nuclear marker with an RFP tag. The green and red fluorescence signals in vegetative hyphae and infectious hyphae were observed by dual fluorescence (Zeiss LSM710, 63× oil).

### 2.11. Statistical Analysis 

Each experiment was performed with three replicates and obtained data were represented as mean ± standard deviation (SD). The significant differences between treatments were statistically determined by one-way analysis of variance (ANOVA) comparison and followed by Duncan’s new multiple-range tests.

## 3. Results

### 3.1. Identification and Expression of MoMAF1

**Figure 1:**
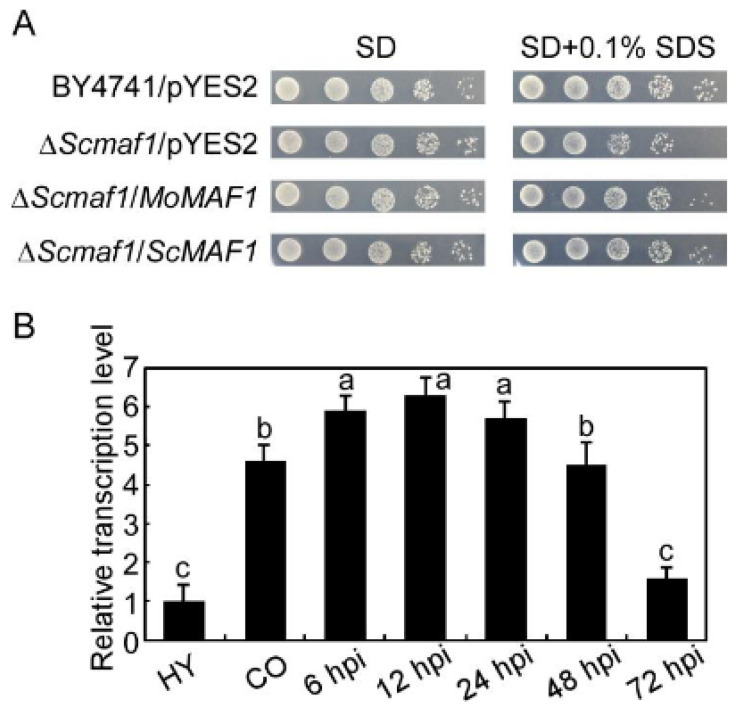
Expression profiles of *MoMAF1* at different developmental stages in *M*. *oryzae* determined by yeast complementation assays: (**A**) *MoMAF1* partially suppressed the growth defect of Δ*Scmaf1* mutant yeast under 0.1% SDS stress. Photographs were taken at 4 days. (**B**) Expression levels of *MoMAF1* at different stages in *M*. *oryzae*. Expression in the hyphal stage was used as an internal reference, and error bars represent the standard deviation (SD). Values on the bars followed by the same letter are not significantly different at (*p* < 0.05).

Examination of the *M. oryzae* genome database revealed that MGG_15675 and *S. cerevisiae* ScMaf1 exhibited high amino acid sequence homology, and we named the MGG_15675 sequence MoMaf1. We first expressed *MoMAF1* in the Δ*Scmaf1* mutant using the yeast expression vector pYES2 and found that Δ*Scmaf1*/*MoMAF1* suppressed the growth defect in sensitivity to SDS in the Δ*Scmaf1* mutant, indicating that MoMaf1 is a functional paralog of ScMaf1 (Figure 1A). In addition, transcription profile analysis of *MoMAF1* at different developmental stages in *M. oryzae* showed that *MoMAF1* was more highly expressed during the infection phase than during the mycelia stage, suggesting that *MoMAF1* participated in the *M. oryzae*–rice interaction (Figure 1B).

### 3.2. MoMAF1-Regulated RNA Synthesis

**Figure 2:**
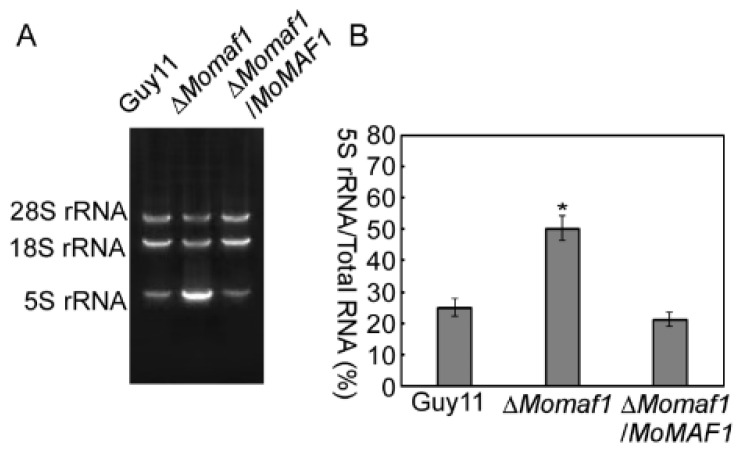
Total-RNA analysis of the test strains: (**A**) The *Magnaporthe oryzae* wild-type strain Guy11, Δ*Momaf1* mutant strain, and Δ*Momaf1/MoMAF1* strain were grown in liquid CM at 28 °C for 36 h and then collected for total-RNA extraction. Total-RNA samples of 5 μg were loaded onto a 1% agarose gel and stained with ethidium bromide before visualization. (**B**) Statistical analysis of the 5S rRNA/total RNA ratio in the different strains. Error bars represent ±SD, and asterisks indicate significant differences (*p* < 0.01). Relative band intensity was quantified by IMAGEJ software. The experiment was repeated three times.

In *Saccharomyces cerevisiae*, Maf1 is a negative regulator of Pol III that represses the synthesis of 5S rRNA and tRNA. Deletion of *MAF1* causes a substantial increase in 5S rRNA and tRNA [23]. To investigate the roles of MoMaf1 in *M. oryzae*, we generated ∆*Momaf1* mutants and verified them by PCR amplification and Southern blot hybridization (Supplementary Figure S1). We then further examined the function of *MoMAF1* in RNA synthesis. RNA extraction successfully yielded one small 5S rRNA species and two large rRNA species (18S and 28S). The ∆*Momaf1* mutant showed dramatically elevated 5S rRNA level compared with that of the wild-type and complemented strain (Figure 2A,B). These results suggested that *MoMAF1* regulated RNA synthesis.

### 3.3. MoMaf1 Was Involved in Vegetative Growth and Conidiation

**Figure 3:**
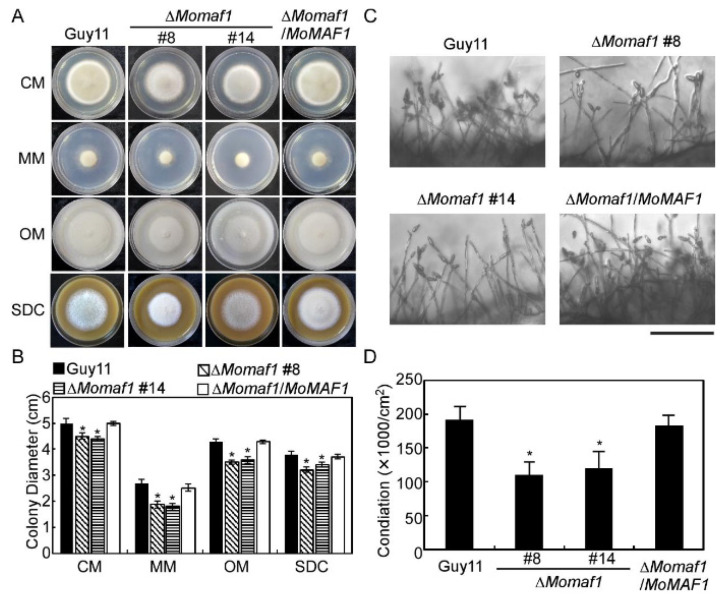
MoMaf1 is involved in vegetative growth and conidiation: (**A**) The Guy11, Δ*Momaf1* mutant, and Δ*Momaf1/MoMAF1* strains were inoculated on CM, MM, OM, and SDC medium; incubated at 28 °C for 7 days; and photographed. (**B**) Statistical analysis of the colony diameter for the strains. Error bars represent ±SD (standard deviation), and asterisks denote statistical significance (*p* < 0.01). (**C**) Conidia were observed and photographed under a light microscope after illumination for 24 h. Scale bar, 50 μm. (**D**) Statistical analysis of the conidial number of the indicated strains. Error bars represent ±SD, and asterisks denote significant differences (*p* < 0.01).

Since we determined that MoMaf1 is a homolog of ScMaf1, we then observed whether the loss of *MoMAF1* in *M. oryzae* led to a considerable defect in vegetable growth. As shown in (Figure 3A,B), destruction of *MoMAF1* leads to defective vegetative growth. Additionally, the Δ*Momaf1* mutant produced fewer conidia than the wild-type strain (Guy11) and the complemented strain (Δ*Momaf1*/*MoMAF1*) (Figure 3C,D). Due to the function of Maf1 in inhibiting transcription, we examined the expression of six conidiation-related genes in the Δ*Momaf1* mutant and Guy11 strain and found that the expression levels of *MoCOM1*, *MoCON2*, *MoHOX2,* and *MoSTUA* were significantly lower in the Δ*Momaf1* mutant, while *MoCOS1* and *MoCON7* levels were not significantly different from those in the Guy11 strain (Supplementary Figure S2), indicating that MoMaf1 was involved in regulating conidiation-related genes.

### 3.4. MoMaf1 Was Required for Penetration and Infectious Growth

**Figure 4:**
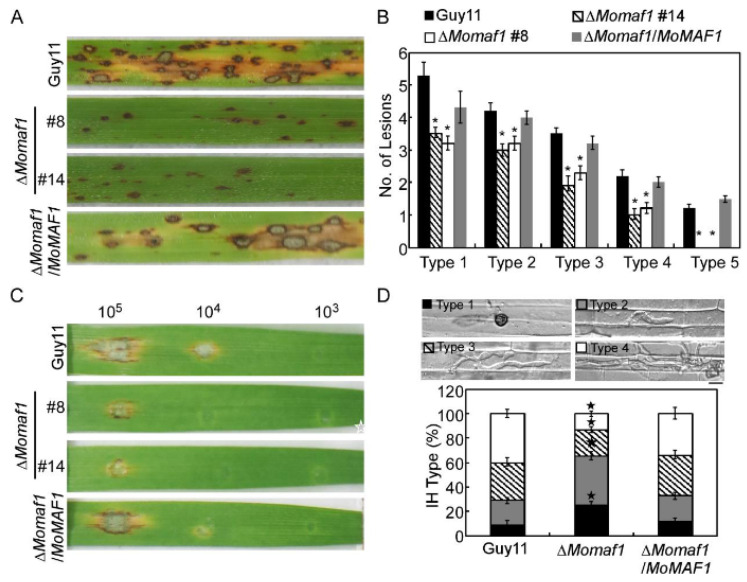
MoMaf1 is required for the virulence of *M. oryzae*: (**A**) Two-week-old rice seedlings (*Oryza sativa* cv. CO-39) were sprayed with 4 mL of a conidial suspension (5 × 10^4^ conidia/mL). The diseased leaves were harvested and photographed at 7 days post-inoculation (dpi). Three independent experiments were performed. (**B**) Quantification of lesions of different types (per 1.5 cm^2^) on diseased rice leaves. Type 1, dark brown pinpoint lesions; type 2, 1.5 mm brown spots; type 3, 2–3 mm lesions with brown margins; type 4, eyespot lesions longer than 3 mm; type 5, coalesced lesions that infected 50% or more of the maximum leaf size (Qian et al., 2021). Error bars represent ±SD, and asterisks denote significant differences (*p* < 0.01). (**C**) Detached barley leaves were drop-inoculated with serial dilutions (1 × 10^5^, 1 × 10^4^, 1 × 10^3^ spores/mL) of conidial suspensions, and the diseased leaves were photographed at 5 dpi. (**D**) Conidial suspensions (1 × 10^5^ spores/mL) were injected into rice sheaths, and infection severity was observed at 36 hpi. The percentages of different types of infectious hyphae (IH) in the rice cells were quantified at 30 hpi. Error bars represent the SD, and asterisks denote the significant differences of each type (*p* < 0.01). Type 1, no penetration; type 2, only a single invasive hypha (IH) without branches; type 3, 1–3 branches but restricted to one cell; type 4, more than three branches and extended to the neighboring cell. Fifty infected cells were observed for each strain and the experiment was repeated 3 times.

To further examine the role of MoMaf1 in virulence, conidial suspensions of the Guy11 strain, the Δ*Momaf1* mutant, and the complemented strain were sprayed onto two-week-old rice seedlings (*Oryza sativa* cv. CO-39). After 7 days of inoculation, the mutant showed reduced virulence, with fewer and smaller lesions on the rice leaves in comparison to the numerous typical lesions caused by the wild-type strain (Guy11) and the complemented strain. A “lesion-type” scoring assay [24] revealed that the numbers of all five types of lesions caused by the Δ*Momaf1* mutant were significantly decreased (Figure 4A,B). Similar results were obtained after the inoculation of conidial suspensions dropped on detached barley leaves, in which the Δ*Momaf1* mutant caused more restricted lesions to form (Figure 4C).

As the Δ*Momaf1* mutant caused fewer and smaller lesions, we further investigated the role of MoMaf1 in penetration and infectious hyphal growth. Statistical analysis of the results showed that approximately 25% of the appressoria formed by the Δ*Momaf1* mutant were unable to penetrate the rice cuticle (type 1), 40% of the penetration sites formed infectious hyphae (IH), but these IH were restricted to one cell with no branches or 1–2 branches (type 2 and type 3, respectively), and less than 15% of the IH extended to the neighboring cells (type 4). In contrast, there were only approximately 10% type 1 and over 70% type 3 and type 4 IH in the Guy11 strain and the complemented strain (Figure 4D). These results indicated that MoMaf1 played a critical role in penetration and infectious growth in rice blast fungus.

### 3.5. MoMaf1 Regulated the Generation of Appressorium Turgor Pressure

Appressoria are critical structures for *M. oryzae* infection. The entire spore can be trafficked into the appressorium, where it undergoes maturation [25]. These coupled processes generate enormous hydrostatic turgor pressure in the appressorium, which has been measured at up to 8.0 MPa, to breach the rice leaf cuticle [26,27]. As appressorium formation in the Δ*Momaf1* mutant was no different from that in the wild-type strain (Supplementary Figure S3), we then examined whether the defect in turgor pressure generation resulted in a reduction in pathogenicity. An appressorium collapse assay was performed to test the appressorial turgor pressure using 1–4 M glycerol solutions [28]. The appressoria of the Δ*Momaf1* mutant exhibited higher collapse ratios than those of the wild-type and complemented strain (Supplementary Figure S4), suggesting that the reduced pathogenicity of the Δ*Momaf1* mutants may be related to the aberrant development of functional appressoria.

### 3.6. MoMaf1 Was Involved in Cell Wall Integrity (CWI)

**Figure 5:**
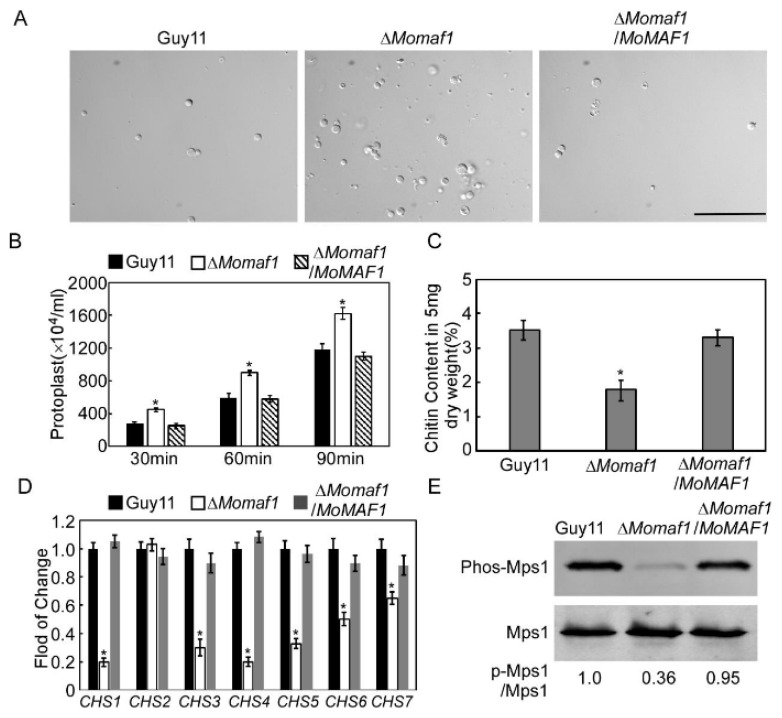
MoMaf1 is involved in cell wall integrity: (**A**) Light microscopic examination of protoplast release after treatment with cell wall-degrading enzymes for 90 min at 30 °C. Scale bar, 50 μm. (**B**) Statistical analysis of the protoplast number. The protoplast number was calculated at 30, 60, and 90 min. Error bars represent ±SD, and asterisks denote significant differences (*p* < 0.01). (**C**) The chitin content was measured in the wild-type Guy11 and Δ*Momaf1* mutant hyphae. The experiment was repeated at least three times with three replicates each time. (**D**) The expression profiles of seven *CHS* genes in the Guy11, Δ*Momaf1* mutant, and complemented strain. (**E**) Total proteins of the Guy11 and Δ*Momaf1* mutant strains were isolated from mycelia to detect the MoMps1 phosphorylation level using the anti-phospho-p44/42 MAP kinase antibody, and the anti-p44/42 MAP kinase antibody was used as a control. The numerical values indicate the ratio of phosphorylated MAPK/endogenous MAPK, and the ratio of phosphorylated MAPK/endogenous MAPK in the Guy11 strain was defined as 1. Relative band intensity was quantified by IMAGEJ software. Three independent experiments that showed similar results were carried out. Asterisks represent signifigant differences (*p* < 0.01).

We further investigated whether MoMaf1 was involved in modulating CWI. First, we assessed the effect of cell wall-degrading enzymes on mycelia in all tested strains [29]. Under the same conditions, the hyphae of the Δ*Momaf1* mutant released more protoplasts than those of the wild-type strain or the complemented strain (Figure 5A,B). Then, we further quantified the chitin that accumulated in the cell wall and found that the Δ*Momaf1* mutant had a higher chitin content than the wild-type strain (Guy11) (Figure 5C). We also examined the expression levels of chitin synthase (*CHS*) genes and found that the expression of six *CHS* genes was significantly reduced in the Δ*Momaf1* mutant, but this was not the case for *CHS2* (Figure 5D). Additionally, the phosphorylation of MoMps1 was clearly decreased when compared with that of the wild-type Guy11 strain (Figure 5E). In addition, the Δ*Momaf1* mutants were more sensitive to the cell wall-perturbing agents CFW and Congo red (CR) (Supplementary Figure S5). Taken together, these results indicated that MoMaf1 was involved in maintaining CWI.

### 3.7. The Subcellular Localization of MoMaf1

**Figure 6:**
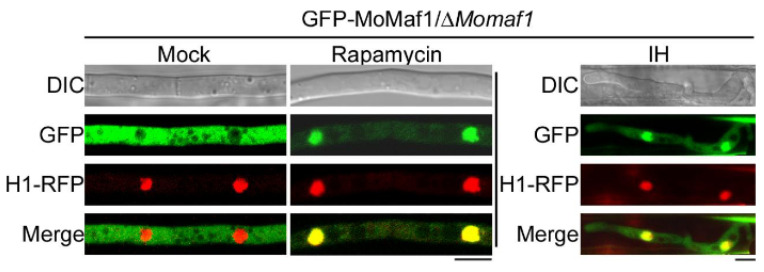
Subcellular localization of GFP-MoMaf1 at different phases. The subcellular localization of GFP-MoMaf1 was observed using the complemented strain with or without rapamycin (30 ng/mL) treatment for 40 min, and nuclei were stained with DAPI (**left**). Scale bar, 10 μm. Subcellular localization of GFP-MoMaf1 during the early infection phase (**right**). Scale bar, 5 μm.

To examine the subcellular localization of MoMaf1, we monitored the GFP-MoMaf1 fusion protein in the wild-type strain under different nutrient conditions. The GFP-MoMaf1 fluorescence signal was localized to the cytoplasm in a nutrient-rich complete medium (CM), and GFP-MoMaf1 was then translocated to the nucleus upon treatment with rapamycin to simulate nitrogen stress (Figure 6). During infection, the GFP fluorescence signal in the cytosol was weaker than that in the nucleus, suggesting that MoMaf1 was transferred from the cytoplasm to the nucleus (Figure 6). These results illustrated that the translocation of MoMaf1 from the cytosol to the nucleus was nutrient-dependent and that tRNA transcription needed to be properly balanced to infect the host.

## 4. Discussion

In eukaryotes, there are three RNA polymerases, designated Pol I, II, and III. Pol II is responsible for the transcription of all protein-coding mRNAs and many non-protein-coding RNAs, while Pol I and Pol III are specialized in the high-level synthesis of non-coding RNA species, rRNA and tRNA, respectively, which are fundamental components of the translation machinery [30]. As the global negative effector of Pol III, Maf1 was originally identified in *S. cerevisiae*, and its orthologues were subsequently characterized in other species [31]. Like previous observations in many species [23,32,33], we have demonstrated in this study that MoMaf1 is also a repressor of Pol III in *M. oryzae*, adding a new member to the Maf1 family.

In this study, we found MoMaf1 was essential for the growth, conidiation, and pathogenicity of *M. oryzae*. As observed in *Candida albicans* and *Fusarium graminearum* [34,35], deletion of *MAF1* both led to a significantly reduced growth rate, indicating that the functions of the Maf1 were conserved. We also found that the Δ*Momaf1* mutant exhibited high sensitivity to cell wall stressors (CFW and CR) and impaired cell wall integrity. In *M. oryzae*, the CWI MAP kinase pathway, consisting of MoMck1, MoMkk1, and MoMps1, was important for appressorium function and virulence [36]. These results indicated that the impaired CWI in Δ*Momaf1* mutant may result in attenuated virulence.

Similar to that of other pathogenic fungi, the infection cycle of *M. oryzae* starts with conidia [37]. The Δ*Momaf1* mutant produced fewer conidia than the wild-type strain, and we also found that the expression of four conidiation-related genes, *MoCOM1*, *MoCON2*, *MoHOX2,* and *MoSTUA,* were significantly reduced in the Δ*Momaf1* mutant, which was consistent with the MoMaf1 protein expression data and indicated that MoMaf1 was involved in sporulation and conidial morphology by regulating the expression of these genes.

In *S. cerevisiae*, several signaling pathways, including those mediated by TOR, modulate the phosphorylation status of Maf1 and mediate various stress signals to Pol III [38]. However, in contrast to the well-studied phosphorylation process of Maf1, little is known about the dephosphorylation of Maf1. By far, studies only revealed that the PP4 complex, with Pph3 as the catalytic subunit, is the major and direct phosphatase of Maf1 [7]. Other PP2A phosphatase complexes and a set of alternative PP2A catalytic subunits that were involved in Maf1 dephosphorylation need further study. 

Importantly, we found that MoMaf1 undergoes cytoplasmic-to-nuclear translocation during infection or in response to nitrogen stress. Similar interaction-dependent translocation has been reported before. Liu and colleagues revealed that MoYvh1 was translocated into the nucleus following oxidative stress to control the maturation of ribosomes, which promoted extracellular protein synthesis and secretion to scavenge rice reactive oxygen species (ROS) [39]. Mature ribosomes carry out extracellular protein synthesis and secretion to scavenge ROS and modulate the rice defense response. Moreover, many transcription factors such as MoMsn2 and MoHac1 both undergo cytoplasmic-to-nuclear translocation when faced with stress or during infection [28,40,41]. So, these studies suggested that a switch of gene expression and protein synthesis was needed for successful infection. In summary, in this study, we identified a pathogenic factor, MoMaf1, which played an important role in growth, conidiation, and pathogenicity in *M. oryzae,* and revealed a novel link between tRNA synthesis and fungal virulence that was mediated by MoMaf1. We concluded that during early infection, *M. oryzae* may need tRNA synthesis to be properly balanced for its pathogenicity. 

## Data Availability

Not applicable.

## Supplementary Materials

The following supporting information can be downloaded at: https://www.mdpi.com/article/10.3390/jof9010106/s1, Figure S1. Gene knockout strategy and southern blot analyses in *M. oryzae*. Figure S2. Transcriptional analysis of six conidiation related genes. Figure S3. Statistical analysis of the appressorium formation rate. Figure S4. MoMaf1 is involved in appressorium turgor generation. Figure S5. MoMaf1 is involved in cell wall stress response. Table S1. Primers used in this study.
