# Supplementary material for: MoMaf1 Mediates Vegetative Growth, Conidiogenesis, and Pathogenicity in the Rice Blast Fungus Magnaporthe oryzae
Source: J Fungi (Basel). 2023 Jan 12;9(1):106. doi: 10.3390/jof9010106 (PMC9861366; doi:10.3390/jof9010106)
Supplement: Supplementary file 1 [file jof-09-00106-s001.zip › jof-2102933-supplementary.pdf]

Table S1. Primers used in this study

Primer name	Sequence (5’-3’)	Remark
MoMaf1 F1:	TAAGTCGACCGAACACTCAGTGCGACTCATG	amplify *MoMAF1* 5’ flank sequence
MoMaf1 F2:	TAAGATATC CTGCACTGCAGGCGAGCCTG	amplify *MoMAF1* 5’ flank sequence
MoMaf1 F3:	TAAACTAGT CATCCTTAGTGTCGCTTTCTGC	amplify *MoMAF1* 3’ flank sequence
MoMaf1 F4:	TAAGAGCTC GGAGAACTTTGTGAAGCGCAC	amplify *MoMAF1* 3’ flank sequence
MoMaf1 BY:	CGAATCGATGAAGATGTAC	amplify *MoMAF1* probe sequence
MoMaf1 ConF:	CCACAGGGCAGGACAAGAAGTTG	amplify *MoMAF1* probe sequence
MoMaf1 ConR:	CTGGTAGCTGAATACGTTGCAG	validation of *MoMAF1* deletion (HPH)
HPH R	GCTGATCTGACCAGTTGCCTA	validation of *MoMAF1* deletion (HPH)
Maf1 Rp27GFP:	TTTCGTAGGAACCCAATCTTCAAAATGGTGAGC AAGGGCGAGGA	*MoMAF1* complementation
Maf1 GFPR:	CTTGTACAGCTCGTCCATGC	*MoMAF1* complementation
qRTCHS1F	TCAACGACGAGGAGAAGCC	qRT-PCR Primer
qRTCHS1R	GTAATCGCAACAGCCAAGA	qRT-PCR Primer
qRTCHS2F	TCCACGACCTTTGCCATCA	qRT-PCR Primer
qRTCHS2R	CGCTTTTGCTTCCGCGACT	qRT-PCR Primer
qRTCHS3F	CGGAAACCAAGGAACAGCG	qRT-PCR Primer
qRTCHS3R	CAGGGAACAACCAAGAACCAC	qRT-PCR Primer
qRTCHS4F	TCGAGGGAAAATGTAACGG	qRT-PCR Primer
qRTCHS4R	TACTGCTGCTGGTGATGGT	qRT-PCR Primer
qRTCHS5F	CCGTGTTGATGGAGGTTGA	qRT-PCR Primer
qRTCHS5R	GATCTGGCGGTCGAGGAAT	qRT-PCR Primer
qRTCHS6F	GAACGGCAGATTTGATGAC	qRT-PCR Primer
qRTCHS6R	ACAAGAGTGCTTCGGTGGC	qRT-PCR Primer
qRTCHS7F	GACATTGAGCTGGAGATTGG	qRT-PCR Primer
qRTCHS7R	CGCCGCTGTTGCTGTTGTT	qRT-PCR Primer
COS1-QRT-F1：	CCCTCAGCCCACATACAACT	qRT-PCR Primer
COS1-QRT-F2：	AGCCTTCGCTCGATACTGAA	qRT-PCR Primer
COM1-QRT-F1：	ACCGATTCTGACGAATCCAG	qRT-PCR Primer
COM1-QRT-F2：	CTGGAACTGCTGTCCTCCTC	qRT-PCR Primer
CON7-QRT-F1：	GCAAGAAGTGCGTTCAAACA	qRT-PCR Primer
CON7-QRT-F2：	TCTCCACTGCTGCCACTATG	qRT-PCR Primer
CON2-QRT-F1：	GGAGCCGAAAACATCAACAT	qRT-PCR Primer
CON2-QRT-F2：	GTTGGTTGGTCCATGCTCTT	qRT-PCR Primer
Hox2-QRT-F1：	CGATAATTGCTCCCACACCT	qRT-PCR Primer
Hox2-QRT-F2：	GAAGGAGTCGGTGGTGACAT	qRT-PCR Primer
StuA-QRT-F1：	CAACATGGGCAGCTCTGATA	qRT-PCR Primer
StuA-QRT-F2：	CCTGCATGCTTTGTAGCGTA	qRT-PCR Primer

MoMaf1 GeneF1:	GCATGGACGAGCTGTACAAGATGAAGGTGC GCATACTTTCCCCAG	*MoMAF1* complementation
MoMaf1 GeneR1:	CACCACCCCGGTGAACAGCTCCTCGCCCTTG CTCACTCACTCAATCTCCATCTGCGCC	*MoMAF1* complementation

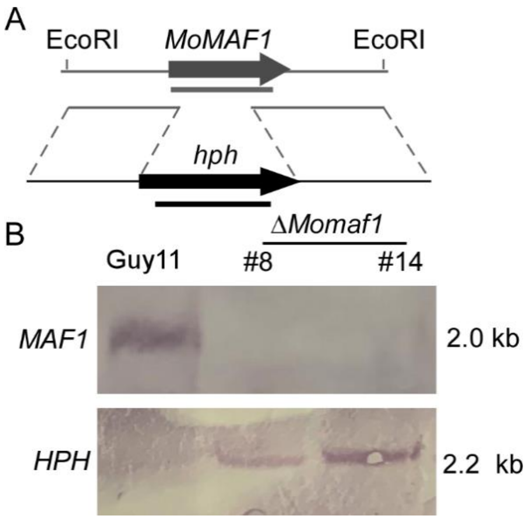

Figure S1. Gene knockout strategy and southern blot analyses in *M. oryzae*. (A)*MoMAF1* gene knock out strategy in *M. oryzae* genome. (B) Southern blot analysis of the *MoMAF1* deletion mutants. The genomic DNA of Guy11 and Δ*Momaf1* mutants were digested with EcoRI and hybridized with *MoMAF1* and *HPH* probe respectively.

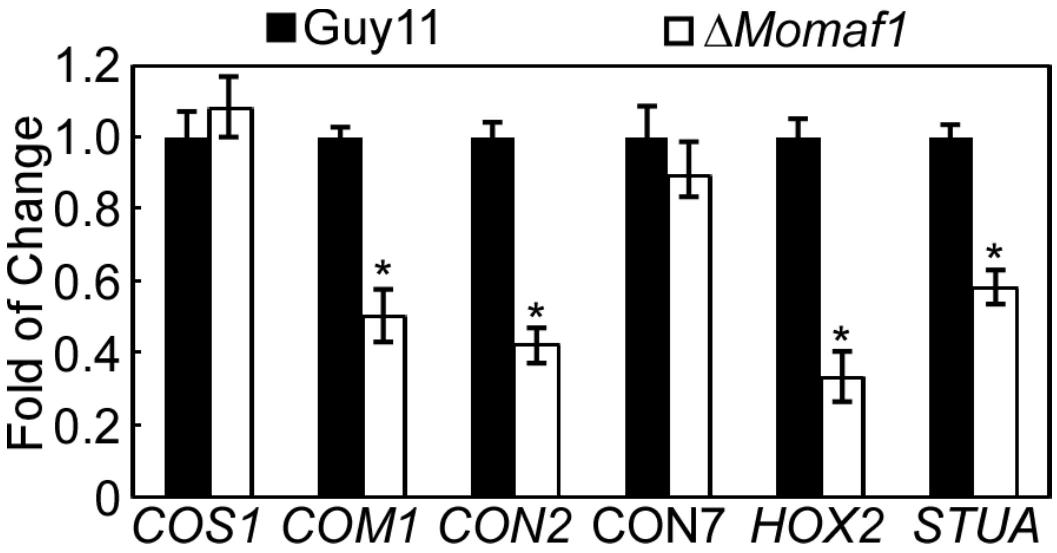

Figure S2. Transcriptional analysis of six conidiation related genes. The expression level of six conidiation related genes in Guy11 and Δ*Momaf1* mutant. Error bars represent ±SD and asterisks represent significant differences ($p < 0.01$).

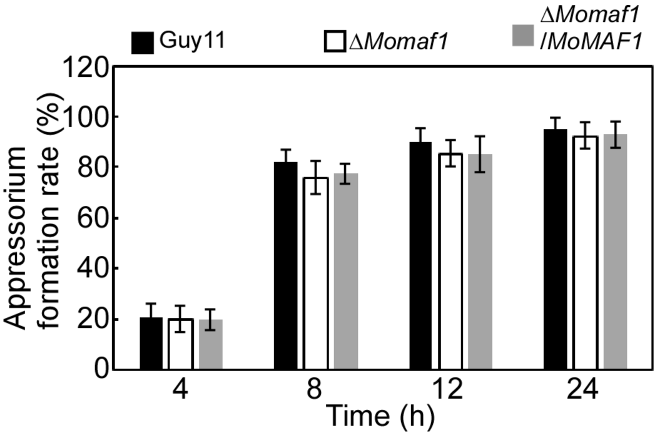

Figure S3. Statistical analysis of the appressorium formation observed at 4, 8, 12 and 24 h time point. Error bars represent the standard deviations. Asterisks represent significant differences (*p*<0.01).

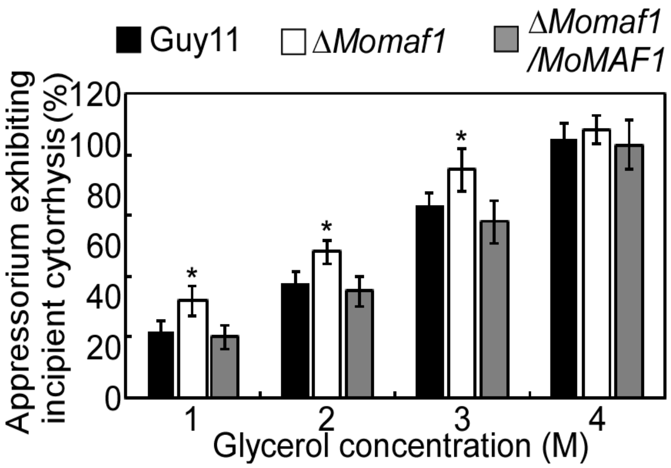

Figure S4. MoMaf1 is involved in appressorium turgor generation. Statistical analysis of the collapsed appressoria onhydrophobic surfaces after 24 h incubation. Error bars represent ±SD and asterisks represent significant differences (*p < 0.01*).

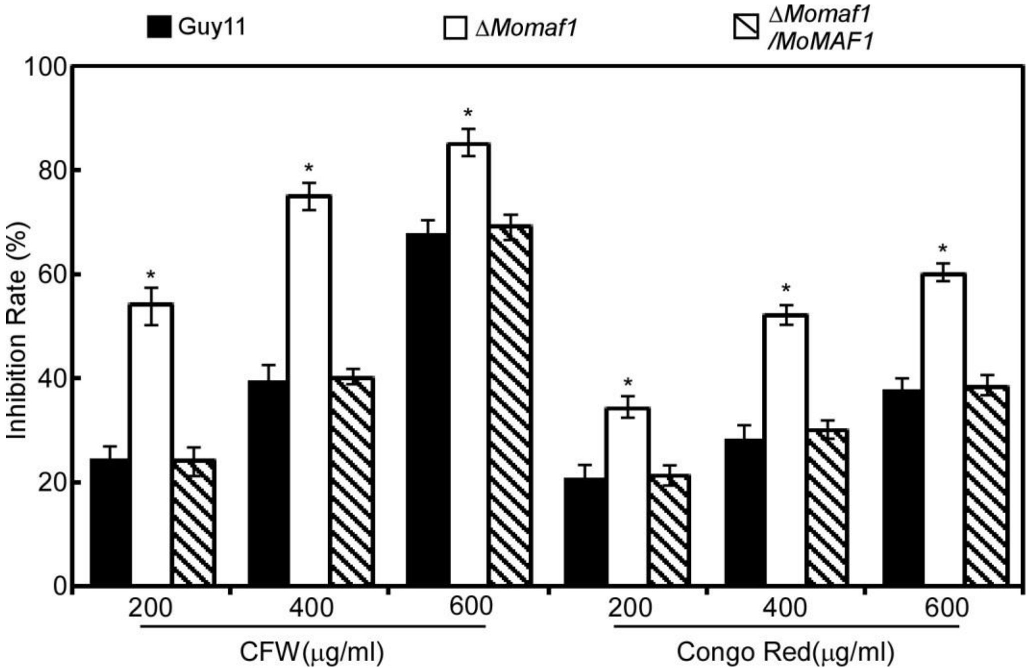

Figure S5. MoMaf1 is involved in cell wall stress response. Guy11, Δ*Momaf1* mutant, and Δ*Momaf1*/*MoMAF1* were incubated on CM plates containing different concentrations of Congo Red (CR) and CFW at 28 C for 7 days. The inhibition rate was determined by plotting the percentage of colonies in the presence of various concentrations of CR and CFW against regular CM. Error bars represent ±SD and asterisks represent significant differences ($p < 0.01$).
